# Triglyceride-glucose index is a predictor of the risk of prostate cancer: a retrospective study based on a transprostatic aspiration biopsy population

**DOI:** 10.3389/fendo.2023.1280221

**Published:** 2024-01-08

**Authors:** Yijie Zhou, Tianqi Li, Guliman Muheiyati, Yajun Duan, Songtao Xiao, Yi Gao, Ning Tao, Hengqing An

**Affiliations:** ^1^ School of Public Health, Xinjiang Medical University, Urumqi, Xinjiang, China; ^2^ School of Traditional Chinese Medicine, Xinjiang Medical University, Urumqi, Xinjiang, China; ^3^ Department of Epidemiological Statistics, School of Public Health, Xinjiang Medical University, Urumqi, Xinjiang, China; ^4^ Department of Urology, The First Affiliated Hospital of Xinjiang Medical University, Urumqi, Xinjiang, China

**Keywords:** prostate cancer, triglyceride-glucose index, insulin resistance, endocrinology, urology

## Abstract

**Background:**

Current research suggests that prostate cancer (PCa), one of the most common cancers in men, may be linked to insulin resistance (IR).Triglyceride-glucose index (TyG index) was made for a marker of insulin resistance. We investigated the relationship between the TyG index and the risk of PCa.

**Objective:**

To assess the correlation and dose-response relationship between TyG index and prostate cancer.

**Method:**

Retrospectively, 316 patients who required prostate biopsy puncture in the First Affiliated Hospital of Xinjiang Medical University from March 2017 to July 2021 were collected, and the relationship between factors such as the TyG index and prostate cancer was analyzed by Logistic regression model combined with a restricted cubic spline.

**Results:**

(1) The differences in age, initial PSA and TyG index between the two groups were statistically significant; (2) Logistic regression results showed that the risk of prostate cancer in the highest quartile of the TyG index (Q4) was 3.387 times higher than that in the lowest quartile (Q1) (OR=3.387,95% CI [1.511,7.593], *P*=0.003); (3) The interaction results showed a significant interaction between the TyG index Q4 group and age with the risk of developing prostate cancer (*P* for interaction<0.001). (4) The results of the restricted cubic spline showed a linear dose-response relationship between the TyG index and the risk of prostate cancer; (5) The Receiver operating characteristic (ROC) curve results showed that the area under the curve (AUC) of the TyG index combined with initial PSA and age was 0.840, with a sensitivity and specificity of 62.5% and 93.3%, respectively.

**Conclusion:**

TyG index and age are risk factors for prostate cancer, and the interaction between the TyG index and different risk factors may increase the risk of prostate cancer. TyG index has some predictive value for the risk of prostate cancer, and the risk of prostate cancer can be reduced by controlling the levels of blood lipids and blood glucose.

## 1 Introduction

According to GLOBOCAN, in 2020 there will be approximately 1.4 million new cases and 375,000 deaths worldwide from prostate cancer (PCa), the second most common cancer in men and the fifth leading cause of cancer death worldwide (1). And in China, the problem of an ageing population is becoming increasingly serious (2, 3), Prostate cancer incidence and mortality rates are on a significant rise (4–9). Prostate cancer has become a common urological tumor in men, posing a serious risk to human health (1–12). Current factors that may influence prostate cancer are age, family history of tumors, genetic mutations, African ancestry, metabolic syndrome, and others (13–16). Metabolic syndrome is characterized by obesity, insulin resistance (IR), hypertension, and hyperlipidemia (17), and several current studies have demonstrated that insulin resistance is associated with prostate cancer, which can affect the development and progression of Pca through a variety of mechanisms, including the inflammatory pathway (Nuclear Factor Kappa B) (NF-κB) and cytokines, and increase the risk of developing prostate cancer (18–22). The current gold standard for the diagnosis of insulin resistance is Euglycemic-Hyperinsulinemic Clamp (23), but it is slightly cumbersome and expensive to use in practice (24). Studies have demonstrated the high sensitivity of the triglyceride-glucose index (TyG index) for identifying IR (25), the TyG index combines triglycerides (TG) and fasting plasma glucose (FPG), which are important in the diagnosis of IR, so the TyG index can be a reliable proxy for the diagnosis of insulin resistance (26, 27). Elevated TG and FPG have been shown to increase the risk of prostate cancer by Arthur R et al (28–30). And many recent studies have demonstrated that the TyG index is closely associated with the occurrence and development of cancer (31), TyG index can be used as a predictor of breast, colorectal, gastric,thyroid and non-small cell lung cancers (32–42). However, no study has been conducted to illustrate the relationship between TyG index and prostate cancer. Therefore, this study aims to illustrate the relationship between TyG index and prostate cancer, to investigate the dose-response relationship between TyG index and prostate cancer and the predictive value of TyG index in prostate cancer.

## 2 Materials and methods

### 2.1 Study subjects

The examination results of 316 patients who underwent prostate biopsy punctures from March 2017 to July 2021 in the First Affiliated Hospital of Xinjiang Medical University were retrospectively collected and patients were used as study subjects. Patients diagnosed with benign prostatic hyperplasia (BPH) according to histopathology and immunohistochemistry in the Department of Pathology of the First Affiliated Hospital of Xinjiang Medical University were used as the control group, and patients diagnosed with prostate cancer were used as the case group.

#### 2.1.1 Inclusion criteria for the control group

① Those diagnosed with BPH between April 2017 and July 2021 based on histopathology and immunohistochemistry; ② Patients who meet the indications for prostate biopsy punctures; ③ First time prostate biopsy puncture performers; ④ People who are able to read, understand and provide consent.

#### 2.1.2 Exclusion criteria for the control group

① Patients with any type of cancer or previous history of cancer; ② Patients with a history of diabetes mellitus, use of glucose-lowering drugs, use of fenofibrate triglyceride-lowering drugs, and a history of hepatic, renal, or other diseases associated with disorders of lipid metabolism; ③ The data examined are incomplete.

#### 2.1.3 Inclusion criteria for the case group

① Patients diagnosed with prostate cancer based on histopathology and immunohistochemistry between April 2017 and July 2021; ② Patients who meet the indications for prostate biopsy punctures; ③ Patients undergoing their first prostate biopsy puncture with a first diagnosis of prostate cancer; ④ Patients with prostate cancer who were able to read, understand, and provide consent forms and complete medical records.

#### 2.1.4 Exclusion criteria for the case group

① Patients with a history of other types of cancer; ② Patients with a history of diabetes mellitus, use of glucose-lowering drugs, use of fenofibrate triglyceride-lowering drugs, and a history of hepatic, renal, or other diseases associated with disorders of lipid metabolism; ③ The data examined are incomplete.

#### 2.1.5 Indications for performing prostate biopsy punctures

① Patients with persistently elevated prostate-specific antigen (PSA) or greater than 4 ng/ml; ② A hard nodule of the patient’s prostate gland was found on physical examination of the prostate gland; ③ Transrectal prostate ultrasound hypoechoic nodules, prostate magnetic resonance abnormal signal nodules; ④ If the patient’s first puncture is negative, but with high-grade PIN, etc., and if the patient’s prostate-specific antigen is persistently elevated, another prostate puncture biopsy may be performed.

### 2.2 Study methods

The study was a case-control study in which demographic data such as age, ethnicity, and education level of the study subjects were obtained based on hospital medical record information, and their family history of cancer was also asked; fasting vena cava blood was drawn from the study subjects to determine their initial serum PSA, fasting TG, FPG, blood calcium, testosterone, blood potassium, total cholesterol(TC), low-density lipoprotein (LDL), etc.

In both groups, blood specimens were collected from 8:00 to 9:00 am on the next day of admission in a fasting state, and were immediately sent for examination, and the results were kept. Patients who met the criteria for prostate biopsy were scheduled for transrectal prostate biopsy the day after the examination. Biopsies were performed by a number of senior urologists using the US BARD biopsy needles using the 12+X puncture method, in which 1-2 needles were punctured as the X-needle for suspicious areas suggested on nuclear magnetic resonance imaging or ultrasound, and the rest of the area was punctured by the systematic 12-needle puncture method (43, 44). Each needle of tissue was individually placed into a fixation vial containing 10% methanol aqueous solution, marked with the hospitalization number and date of puncture, and uniformly sent to the Department of Pathology of the First Affiliated Hospital of Xinjiang Medical University for examination, where the histopathological results were recorded and diagnosed by a senior pathologist.

#### 2.2.1 Diagnostic criteria and index definitions

TyG index was calculated from the formula: TyG = LN (fasting TG [mg/gl] * FPG [mg/gl]/2) (45). Body mass index (BMI) = weight (kg)/[height (m)]^2 (46). According to the World Health Organization’s criteria for smoking, divided into smokers and non-smokers. Alcohol consumption was also divided into drinkers and non-drinkers according to WHO criteria.

#### 2.2.2 Statistical analysis

The data were analyzed using SPSS 26.0 and R 4.0.5 software. Use nonparametric tests, t-test, and restricted cubic spline plots to test the distribution of continuous data.

Normally distributed data are presented as means (standard deviations), while nonparametric data are expressed as median, minimum and maximum values. Categorical data were expressed as percentages, and the chi-square test was used for comparison between groups. The TyG index was divided into four quartiles according to the interquartile spacing method (‘Q1’ is<8.389, ‘Q2’ is 8.389-8.805, ‘Q3’ is 8.805-9.241, ‘Q4’ is>9.241), and the odds ratio (OR) of each quartile was calculated using the first quartile as a reference, and logistic regression was used to calculate the OR of the TyG index and prostate cancer. Logistic regression was used to develop correlation models to test the correlation between prostate cancer and the respective variables; model 1 was unadjusted, model 2 was adjusted for age and initial PSA, and model 3 was adjusted for age, initial PSA, smoking history, alcohol consumption, family history of cancer, BMI, TC, and LDL. Logistic regression was used to analyze the interaction between the TyG index and age and initial PSA. The test level α = 0.05. The linearity of the dose-response curves was assessed using restricted cubic spline plots and logistic regression models. The Receiver operating characteristic (ROC) curve were applied to assess the predictive value of the TyG index for prostate cancer.

## 3 Results

### 3.1 Baseline characteristics

A total of 316 individuals were included. Among them, 136 were in the case group with an age of (70.73 ± 9.80) years. The control group consisted of 180 individuals aged (65.10 ± 8.51) years. The differences in age, initial PSA and TyG index between the two groups were statistically significant (*P*<0.05) **Table 1**.

**Table 1:** Baseline characteristics of study participants in general.

Variable	Prostate cancer patients (n=136)	People with benign prostatic hyperplasia (n=180)	χ^2^/t/w ratio	*P*
Age/[ $\bar{x}\pm s$ ,years]	70.73 ± 9.80	65.10 ± 8.51	-5.345	<0.001
Education level/case (%)			2.989	0.224
Elementary school and below	29(21.30)	26(14.40)		
Middle school including technical secondary school	68(50.00)	104(57.80)		
College and above	39(28.70)	50(27.80)		
Domicile/case (%)			0.001	0.976
City	112(82.40)	148(82.20)		
Countryside	24(17.60)	32(17.80)		
Marital Status/case (%)			<0.001	>0.999
Married	132(97.10)	174(96.70)		
Other	4(2.90)	3(3.30)		
Nationality/case (%)			1.363	0.243
Han	98(72.10)	140(77.80)		
Other	38(27.90)	40(22.20)		
Family history of cancer/case (%)			0.430	0.512
Yes	8(5.90)	14(7.80)		
No	128(94.10)	166(92.20)		
Smoking history/case (%)			1.241	0.265
Yes	36(26.50)	38(21.10)		
No	100(73.50)	142(78.90)		
History of alcohol consumption/case (%)			0.088	0.767
Yes	15(11.00)	18(10.00)		
No	121(89.00)	162(90.00)		
Testosterone/[*M*(*P*_75_,*P*_25_),nmol/L]	15.06(21.08,9.04)	15.68 (21.18,10.18)	0.952	0.342
LDL/[*M*(*P*_75_,*P*_25_),mmol/L]	2.34 (3.10,1.58)	2.44 (3.33,1.55)	1.112	0.267
Blood potassium/[*M*(*P*_75_,*P*_25_),mmol/L]	3.71 (4.16,3.26)	3.67 (4.07,3.27)	-0.820	0.413
TyG Index[ $\bar{x}\pm s$ ]	8.93 ± 0.69	8.74 ± 0.58	-2.751	0.006
TyG Index grouping			8.228	0.042
Q1(<8.389)	28 (20.60)	52 (28.90)		
Q2(8.389-8.805)	32 (23.50)	46 (25.60)		
Q3(8.805-9.241)	32 (23.50)	48 (26.70)		
Q4(>9.241)	44 (32.40)	34 (18.90)		
Initial PSA/ [*M*(*P*_75_,*P*_25_),ng/mL]	25.60(147.33,11.31)	8.10(12.58,6.27)	-9.160	<0.001
BMI/[*M*(*P*_75_,*P*_25_),kg/m^2^]	24.00(26.00,21.00)	24.00(26.00,21.00)	-0.403	0.687
ALP/[IU/L]	71.12(98.29,59.48)	71.35 (84.50,61.35)	-0.385	0.700
Blood Calcium/[*M*(*P*_75_,*P*_25_),mmol/L]	2.27(2.33,2.19)	2.29(2.37,2.19)	-0.600	0.549
TC[*M*(*P*_75_,*P*_25_),mmol/L]	4.01(4.68,3.48)	4.26(4.78,3.33)	-0.570	0.569

TyG, index triglyceride-glucose index; Initial PSA, initial prostate-specific antigen; TC, total cholesterol; LDL, low-density lipoprotein; BMI, body mass index; ALP, alkaline phosphatase.

### 3.2 Logistic regression analysis of factors affecting prostate cancer

The results showed that in model 1, the OR (95% CI) for prostate cancer was 1.000,1.292 (0.679-2.460),1.238 (0.652-2.351), and 2.403 (1.266-4.564) with increasing quartiles of TyG index (*P* for trend=0.012). In model 2, factors associated with the risk of developing prostate cancer included age (OR=1.056,95% CI [1.020,1.094], *P*=0.002), initial PSA (OR=1.059,95% CI [1.031,1.088], *P*<0.001), and TyG index (*P* for trend=0.001), the risk of prostate cancer in the highest quartile of the TyG index (Q4) was 3.387 times higher than that in the lowest quartile (Q1) (OR=3.387,95% CI [1.511,7.593], *P*=0.003). In model 3, the OR (95% CI) for prostate cancer was 1.000,0.886 (0.350-2.241),2.065 (0.874-4.878), and 2.854 (1.200-6.790) as increasing quartiles of the TyG index (*P* for trend=0.002) **Table 2**.

**Table 2:** Logistic regression analysis of factors affecting prostate cancer.

Variable	Model 1		Model 2		Model 3	
	OR (95%CI)	*P* Value	OR (95%CI)	*P* Value	OR (95%CI)	*P* Value
TyG Index (mmol/L)		0.012^a^		0.001^a^		0.002^a^
Q1(<8.389)	1.000	0.045	1.000	0.006	1.000	0.012
Q2(8.389-8.805)	1.292(0.679-2.460)	0.436	1.041(0.437-2.479)	0.927	0.886(0.350-2.241)	0.737
Q3(8.805-9.241)	1.238(0.652-2.351)	0.514	2.064(0.919-4.636)	0.079	2.065(0.874-4.878	0.112
Q4(>9.241)	2.403(1.266-4.564)	0.007	3.387(1.511-7.593)	0.003	2.854(1.200-6.790)	0.011
Age (years)			1.056 (1.020-1.094)	0.002	1.071 (1.031-1.113)	<0.001
Initial PSA(ng/mL)			1.059(1.031-1.088)	<0.001	1.070(1.039-1.102)	<0.001
Smoking history					1.635(0.779-3.429)	0.194
alcohol consumption					0.527(0.169-1.649)	0.271
Family history of cancer					1.095(0.344-3.482)	0.878
BMI(kg/m2)					1.128(1.023-1.243)	0.016
TC(mmol/L)					0.857(0.706-1.039)	0.116
LDL(mmol/L)					1.058(0.709-1.578)	0.783

TyG, index triglyceride-glucose index; Initial PSA, initial prostate-specific antigen; TC, total cholesterol; LDL, low-density lipoprotein; BMI, body mass index.

Model 1 was unadjusted. Model 2 was adjusted for age and initial PSA. Model 3 adjusted for age, initial PSA, smoking history, alcohol consumption, family history of cancer, BMI, TC, LDL.

a P for trend.

### 3.3 Effect of TyG index interacting with different risk factors on prostate cancer

The interaction results showed a significant interaction between the TyG index Q4 group and age with the risk of developing prostate cancer (*P* for interaction<0.001). Tests for the interaction between TyG index Q2 group (*P* for interaction=0.079), Q3 group (*P* for interaction=0.077), and age and risk of prostate cancer, as well as TyG index Q3 group (*P* for interaction=0.100) and initial PSA and risk of prostate cancer, were not significant **Table 3**.

**Table 3:** Effect of interaction between age, initial PSA and TyG index on prostate cancer.

		Initial PSA(ng/mL)	*P* for interaction	Age (years)	*P* for interaction
TyG Index, mmol/L (in quartile)	Q1(n=80)(<8.389)	1.000	0.079	1.000	0.005
	Q2(n=78)(8.389-8.805)	1.106(1.019-1.200)	0.016	1.008(0.999-1.018)	0.079
	Q3(n=80)(8.805-9.241)	1.076(0.986-1.174)	0.100	1.009(0.999-1.018)	0.077
	Q4(n=78)(>9.241)	1.118(1.016-1.231)	0.022	1.018(1.008-1.027)	<0.001

TyG, index triglyceride-glucose index; Initial PSA, initial prostate-specific antigen.

### 3.4 Dose-response relationship between TyG index and prostate cancer

**Figure 1:**
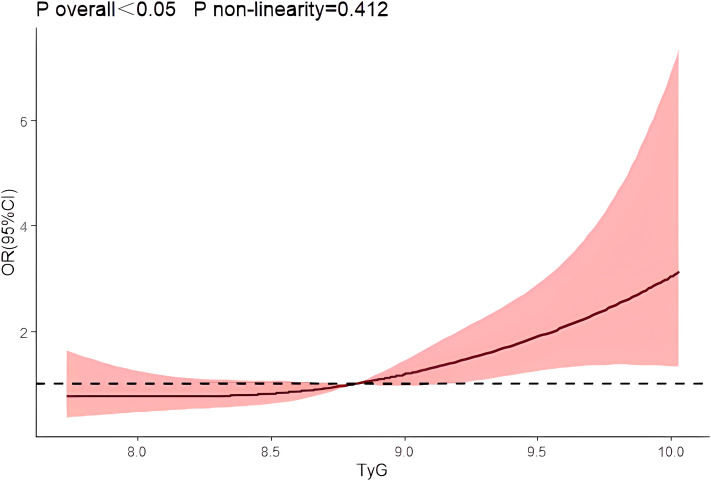
Dose-response relationship between TyG index and prostate cancer.

After using restrictive cubic splines and adjusting for relevant confounders, there was a linear dose-response relationship between the TyG index and risk of prostate cancer prevalence (*P* overall<0.05, *P* non-linearity=0.412) **Figure 1**.

### 3.5 Predictive value of TyG index, initial PSA, and age on the risk of prostate cancer development

**Figure 2:**
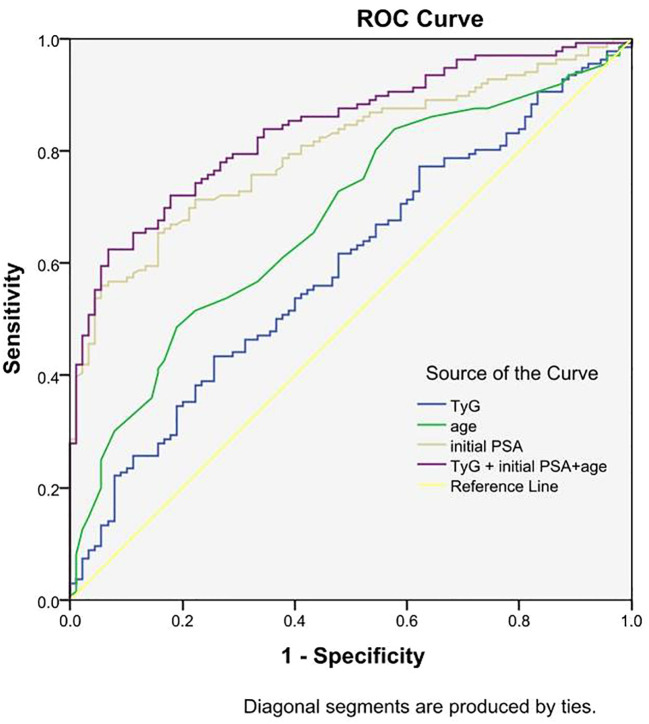
Predictive value of TyG index, initial PSA, and age on the risk of prostate cancer.

ROC curves for predicting prostate cancer were plotted based on the TyG index, initial PSA, and age. The results showed that the area under the curve (AUC) of TyG index, initial PSA, and age were 0.539, 0.801, and 0.680, respectively, while the AUC of TyG index combined with initial PSA and age was improved to 0.840, with a sensitivity and specificity of 62.5% and 93.3%, respectively. The accuracy of the TyG index combined with initial PSA and age in predicting the risk of prostate cancer was high. See **Table 4** and **Figure 2**.

**Table 4:** ROC curve analysis of TyG index, age, initial PSA, and all three in combination with prostate cancer.

Variables	AUC	95% CI	*P*	Sensitivity	Specificity	Youden’s index	Truncation value
TyG Index	0.593	0.530-0.657	0.005	0.434	0.744	0.178	9.091
Initial PSA	0.801	0.750-0.852	<0.001	0.559	0.944	0.503	21.310
Age	0.680	0.620-0.740	<0.001	0.485	0.811	0.296	71.500
TyG Index+Initial PSA+Age	0.840	0.795-0.886	<0.001	0.625	0.933	0.588	0.456

TyG, index triglyceride-glucose index; Initial PSA, initial prostate-specific antigen.

## 4 Discussion

In this retrospective study, we investigated the effect of the TyG index on the risk of developing PCa and found for the first time that the TyG index predicts the risk of developing PCa. A meta-analysis showed a strong association between TyG index and cancer development (31),Panigoro S S’s study proved that there was a nonlinear dose-response relationship between TyG index and breast cancer (42), but so far no study has illustrated the dose-response relationship between TyG index and the risk of prostate cancer, and the restricted cubic spline model can intuitively describe the relationship between the independent variable and the dependent variable, therefore, the present study applies the restricted cubic spline model to analyze the relationship between the TyG index and the risk of prostate cancer. The results showed that there was a linear dose-response relationship between the TyG index and the risk of prostate cancer, and Logistic regression analysis showed that the risk of prostate cancer in the TyG index Q4 group was 3.387 times higher than that in the Q1 group, suggesting that the TyG index has an important effect on the occurrence and development of prostate cancer. We also used Logistic regression model to analyze the effect of interaction between age, initial PSA and TyG index groups on prostate cancer, and the results showed that there was an interaction between TyG index Q4 group and age and initial PSA, and the interaction between TyG index and different risk factors may increase the risk of prostate cancer. Therefore, we further analyzed the predictive value of TyG index and age on the risk of prostate cancer using ROC curves, and found that the AUCs of TyG index, initial PSA, and age were 0.539, 0.801, and 0.680, respectively. Whereas the AUC of TyG index combined with initial PSA and age was improved to 0.840, with a sensitivity and specificity of 62.5% and 93.3 percent. It indicates that TyG index combined with initial PSA and age has better accuracy than age, initial PSA and TyG index alone in predicting the risk of prostate cancer.

Studies by Albanes D et al. demonstrated that insulin resistance is associated with prostate cancer (20, 47), while Lebovitz HE et al. demonstrated that insulin resistance is associated with such as hyperinsulinemia (48), IGF levels (19, 20), and Phosphatidylinositol 3-kinase (PI3K)/protein kinase B (Akt) signaling pathways (49), which may play an important role in the development of prostate cancer. In patients with insulin resistance, insulin sensitivity is reduced, the efficiency of glucose uptake and utilization decreases, and the body compensatorily secretes excess insulin, inducing the development of hyperinsulinemia (50). Hyperinsulinemia has been shown to have a direct effect on the liver, inhibiting the production of insulin-like growth factor-binding proteins 1 and 2 (IGFBP-1,-2), while stimulating the production of insulin-like growth factor-1 (IGF-1) and increasing the bioavailability of IGF-1 (51–53). Whereas binding of IGF-1 to the IGF receptor activates the p21 ras/mitogen-activated protein kinase (MAPK) pathway and PI3K/Akt pathway (54). Activation of the MAPK pathway causes activated extracellular signal-regulated kinase (ERK) to translocate to the nucleus, reverse transcription activates transcription factors, alters gene expression, promotes cell growth, differentiation and mitosis, and facilitates PCa cell proliferation (55). Activation of the PI3K/Akt pathway promotes the phosphorylation of BAD that a pro-apoptotic Bcl2 family member in cells, phosphorylation of BAD inhibits the ability of BAD to bind and constrain the anti-apoptotic Bcl2 family members that BclxL and Bcl2, leading to apoptosis resistance in PCa cells, which in turn causes prostate cancer (54, 56). Increased bioavailability of IGF-1, increased binding of IGF-1 and IGF receptors, and increased intensity of IGF-1 action predispose to the induction of prostate cancer.

The TyG index, calculated from fasting triglycerides and blood glucose, was initially proposed as a biomarker of insulin resistance (25, 45), and subsequent studies by Tutunchi H and Lv L et al. have shown it to be associated with diabetes mellitus (57), hepatic fibrosis (58), cardiovascular disease (59), and erectile dysfunction (60). Blood glucose and lipids are important components of the TyG index, and some studies have reported a strong association between them and the development of prostate cancer (61–63). Aljada A et al. showed that hyperglycemia leads to an increase in intranuclear NF-κB in human subjects, that two of the target genes of NF-κB, such as cell cycle protein D1 and cMyc, play important roles in cell growth and proliferation, and that NF-κB itself regulates many genes involved in the process of cell proliferation, tumor formation, and metastasis, thereby influencing cancer development (61, 62). Elevation of body lipids causes enhanced lipid metabolism, which in turn causes enhanced adipocyte metabolism (63). Adipocytes produce inflammatory cytokines and form a protumorigenic environment (64), and periprostatic adipocytes also promote the extracapsular expansion of prostate cancer through chemokines, which affect the development of prostate cancer (65). In addition, increased fat cell metabolism also increases leptin production, a hormone secreted from fat cells that suppresses appetite and increases basal metabolism through metabolic signaling. It has been suggested that high leptin levels are associated with prostate, colon, breast, and endometrial cancers and that leptin may play a role in this through cell proliferation via MAPK signaling. On the other hand, it has also been suggested that leptin may stimulate angiogenesis, increase matrix metalloproteinase-2 expression, and lead to cancer metastasis (66). In conclusion, elevated blood glucose and lipids can affect the development of prostate cancer through a variety of mechanisms, and there is a correlation between TyG index and prostate cancer.

There are certain limitations in this study: First, this study is a single-center case-control study, there are some limitations in the selection of samples, only some of the influencing factors have been collected, and there is a lack of data on patients from different geographical regions, pending the expansion of the sample size at a later stage and the collection of more comprehensive factors for a multicenter study. Secondly, this study is a cross-sectional survey, only investigated a certain point in time indicators, did not take into account the influence of the trend of the indicators on the results, and the TyG index will be affected by diet-related factors, later we will use this study as a baseline, and carry out a cohort study to further observe the impact of changes in the TyG index on the risk of prostate cancer.

In conclusion, the TyG index is an important influencing factor for prostate cancer and has some predictive value for the risk of prostate cancer, which needs to be further determined in future prospective studies.

## 5 Conclusions

This study found that the TyG index is a risk factor for prostate cancer, and the interaction between the TyG index and different risk factors may increase the risk of prostate cancer. There is a linear dose-response relationship between the TyG index and the risk of prostate cancer, and the TyG index has a certain predictive value of the risk of prostate cancer, and the risk of prostate cancer can be reduced by controlling the levels of blood lipids and blood glucose. By controlling blood lipid and blood glucose levels, the risk of prostate cancer can be reduced.

## Ethics approval and consent to participate

The study involving human participants. The human data used in this study were obtained and applied in accordance with the Declaration of Helsinki. The study was reviewed and approved by the Ethics Committee of First Affiliated Hospital of Xinjiang Medical University (approval number: 20220308-166). Subjects provided written informed consent for participation in this study.

## Data availability statement

The original contributions presented in the study are included in the article/**Supplementary Material**. Further inquiries can be directed to the corresponding authors.

## Ethics statement

The studies involving humans were approved by Ethics committee of First Affiliated Hospital of Xinjiang Medical University. The studies were conducted in accordance with the local legislation and institutional requirements. The participants provided their written informed consent to participate in this study. Written informed consent was obtained from the individual(s) for the publication of any potentially identifiable images or data included in this article.

## Author contributions

YZ: Methodology, Software, Writing – original draft, Writing – review & editing. TL: Formal analysis, Methodology, Project administration, Validation, Writing – review & editing. GM: Conceptualization, Data curation, Investigation, Writing – review & editing. YD: Investigation, Methodology, Software, Validation, Visualization, Writing – review & editing. SX: Formal analysis, Methodology, Supervision, Visualization, Writing – review & editing. YG: Conceptualization, Project administration, Resources, Writing – review & editing. NT: Data curation, Formal analysis, Methodology, Project administration, Resources, Supervision, Validation, Visualization, Writing – review & editing. HA: Conceptualization, Funding acquisition, Investigation, Resources, Software, Visualization, Writing – review & editing.
